# Mobile Phone Use and its Association With Sitting Time and Meeting Physical Activity Recommendations in a Mexican American Cohort

**DOI:** 10.2196/mhealth.4926

**Published:** 2016-06-16

**Authors:** Matthew Chrisman, Wong-Ho Chow, Carrie R Daniel, Xifeng Wu, Hua Zhao

**Affiliations:** ^1^ The University of Texas MD Anderson Cancer Center Department of Epidemiology Houston, TX United States

**Keywords:** mobile phone, physical activity, Mexican Americans, sedentary lifestyle

## Abstract

**Background:**

The benefits of physical activity (PA) are well-documented. Mobile phones influence PA by promoting screen-based sedentary time, providing prompts or reminders to be active, aiding in tracking and monitoring PA, or providing entertainment during PA. It is not known how mobile phone use is associated with PA and sitting time in Mexican Americans, and how mobile phone users may differ from nonusers.

**Objective:**

To determine the associations between mobile phone use, PA, and sitting time and how these behaviors differ from mobile phone nonusers in a sample of 2982 Mexican-American adults from the Mano a Mano cohort.

**Methods:**

Differences in meeting PA recommendations and sitting time between mobile phone users and nonusers were examined using chi-square and analysis of variance tests. Logistic regression was used to examine associations between mobile phone use, PA, and sitting.

**Results:**

Mobile phone users were more likely to be obese by body mass index criteria (≥30 kg/m^2^), younger, born in the United States and lived there longer, more educated, and sit more hours per day but more likely to meet PA recommendations than nonusers. Males (odds ratio [OR] 1.42, 95% CI 1.16-1.74), use of text messaging (OR 1.26, 95% CI 1.03-1.56), and having a higher acculturation score (OR 1.27, 95% CI 1.07-1.52) were associated with higher odds of meeting PA recommendations. Sitting more hours per day was associated with being male, obese, born in the United States, a former alcohol drinker, and having at least a high school education. Among nonusers, being born in the United States was associated with higher odds of more sitting time, and being married was associated with higher odds of meeting PA recommendations.

**Conclusions:**

Mobile phone interventions using text messages could be tailored to promote PA in less acculturated and female Mexican American mobile phone users.

## Introduction

The importance of physical activity (PA) for health is well-documented [1], yet evidence shows that Hispanic people in the United States have higher rates of inactivity than other ethnicities and are less likely to meet US national PA recommendations [2]. Additionally, increasing evidence suggests that more time spent sitting leads to detrimental health effects [3], thus interventions are needed to increase PA and reduce sitting time.

Mobile phone interventions are one potential avenue for promoting activity and reducing sitting [4,5]. Mobile devices have become ubiquitous, with 6.7 billion mobile phone subscriptions worldwide at the beginning of 2014 [6]. As of 2012, mobile phone ownership among US Hispanic adults was 86%, a proportion similar to non-Hispanic whites (84%) and blacks (90%) [7]. The small size and ease of use of mobile devices mean they can be carried anywhere and ultimately may influence behavior through sedentary “screen time,” while texting, emailing, or playing games, or using the devices while standing or participating in moderate activity [8]. They can also provide an impetus for being active through apps that provide reminders or prompts for participating in PA and may aid in tracking and monitoring amounts of activity one engages in, particularly because they were designed to be used while being mobile [8].

Several recent reviews have showed the value in using mobile phones in PA interventions [9,10]. In particular, studies using mobile phones reported increases in PA and reductions in inactivity in a variety of settings and populations [9,10]. However, to prepare for such an intervention, it is helpful to know the extent of any group differences between mobile phone users and nonusers and what associations, if any, exist between mobile phone use and activity behaviors. The relationships between mobile phone use, PA, and sitting time have not been well-described, particularly among Hispanic people in the United States. To better understand PA and sitting time, mobile phone use appears to be a variable worthy of exploring in this population [8]. Additionally, considering that mobile phone owners are more likely to be younger, be college graduates, and have a higher income [11], there may be important behavioral or sociodemographic differences that affect health between users and nonusers of mobile phones.

Hispanic people in the United States are predominantly of Mexican descent [12], and knowing more about their PA and sitting time behaviors may be informative for this group as a whole. The Mexican American Mano a Mano Cohort study is a population-based study of more than 25,000 participants of Mexican origin who have provided data on their activity and sitting behavior, including a subset of participants who have offered data on their usage of mobile phone devices. Because this population has a high rate of mobile phone ownership, this cohort provides an opportunity to examine relationships among these factors. This study’s purpose was therefore to determine the associations between mobile phone use, PA, and sitting time and how these behaviors differ from mobile phone nonusers in a sample of urban-dwelling Mexican American adults from the Mano a Mano cohort. To our knowledge, ours is the first report to examine these associations in this population. Findings can be used to tailor future interventions in this area.

## Methods

### Recruitment

Mano a Mano is an ongoing prospective study examining cancer and chronic disease risk factors in adults residing in the Houston metropolitan area of Texas. Details of this cohort are described elsewhere [13]. Briefly, the study was initiated in 2001 and currently enrolls more than 25,000 adults of Mexican origin. The mean age is 40.8 years (SD 14.2), 73.7% were born in Mexico, and 79.3% are female. Data collection spans a range of social and health characteristics, and trained interviewers administer questionnaires at the participants' homes in their preferred language (English or Spanish). This study used a subset of the overall Mano a Mano cohort. Participants providing data for this study were recruited beginning in 2012, when questions on media use, including mobile phones, were first asked. The institutional review board at The University of Texas MD Anderson Cancer Center approved all study procedures.

### Data

Sociodemographic characteristics assessed at baseline include age, sex, marital status, education, birthplace (Mexico or United States), body mass index (BMI), acculturation, alcohol drinking and smoking status (current, former, and never), and years lived in the United States. Age was grouped into categories of 21-39, 40-54, and 55+ years to account for the potential nonlinear relationship with PA [14]. Marital status was categorized as married or not married, and education was categorized as some high school or less or high school graduate and beyond. Body mass index was calculated from measured height and weight and participants were categorized as obese (BMI ≥ 30 kg/m^2^) or not obese (BMI <30 kg/m^2^). Degree of acculturation was assessed using the Bidimensional Acculturation Scale for Hispanics [15], which measures linguistic preference when speaking, watching television, listening to the radio, and reading. Responses ranged from 1-4 and were dichotomized into low (≤2) or high (>2), with high values reflecting a preference for and fluency in English and a high degree of acculturation. This variable was dichotomized to maintain consistency with other reports using this cohort [16,17].

Physical activity was assessed using the International Physical Activity Questionnaire (IPAQ) short form, which has been validated in multiple populations and exhibits acceptable reliability in Mexican Americans [18,19]. The IPAQ measures activity bouts of at least 10 minutes by domain (work, home, transportation, and recreation) in the past week, and PA is classified by intensity (vigorous, moderate, walking) [20]. Vigorous and moderate activity were used to calculate whether the participants met the US national PA recommendations, which state that individuals should engage in 150 minutes of at least moderate-intensity activity each week, with 1 minute of vigorous PA considered equivalent to 2 minutes of moderate PA [1]. A single item on the IPAQ assessed time spent sitting on a normal weekday in the past 7 days, which exhibits acceptable reliability in adults [21]. Sitting time was dichotomized into 0-3 hours per day and greater than 3 hours per day, which was done to maintain consistency with other reports in the literature [16,22].

Media use was assessed by asking whether participants use a mobile phone; if they use it for playing music, sending text messages, accessing the Internet, social media use, and sending and receiving email; and how often they use it for those functions.

### Statistical Analysis

Physical activity and sitting data were computed according to published guidelines [20]. Descriptive statistics were examined for all variables, and differences between mobile phone users and mobile phone nonusers were examined using chi-square and analysis of variance tests. Analyses were stratified based on the a priori hypothesis that mobile phone users and nonusers are different. Unadjusted odds ratios were calculated for PA and sitting time among each sociodemographic variable for mobile phone users and nonusers separately. Multivariate logistic regressions were then conducted to examine odds of meeting US PA recommendations and sitting 3 or more hours per day, controlling for factors that were significant in univariate analyses. All analyses were conducted using SPSS version 22 (IBM Corp., Armonk, NY), and a 2-sided *P* value of .05 was considered statistically significant.

## Results

Overall, there were 2892 participants, of whom 2556 (88.4%) reported using a mobile phone. Compared with nonusers, mobile phone users were more likely to be obese, younger, born in the United States, more educated, more acculturated, meet US PA recommendations but also sit more than 3 hours per day, and live longer in the United States (Table 1). Data on media use types had high percentages of missing data, as follows: playing music (70.5% missing), accessing the Internet (57.7% missing), social media use (73.0% missing), and sending and receiving email (75.2% missing). Thus, these data were not included in regression analyses.

Tables 2 and 3 show the odds of meeting PA recommendations and for sitting more than 3 hours per day among mobile phone users and nonusers separately. Among both mobile phone users and nonusers, more variables were associated with time spent sitting than with meeting PA recommendations. For mobile phone users, younger age, males, higher education, having a high acculturation score, being a current alcohol drinker, and use of text messaging were associated with meeting PA recommendations; whereas, males, born in the United States, not married, higher education, being obese, having a high acculturation score, current or former alcohol drinker, current or former smoker, and use of text messaging were associated with sitting 3 or more hours per day. For mobile phone nonusers, only being married was associated with meeting PA recommendations; whereas, being male, born in the United States, more educated, having a high acculturation score, and current or former smoker were associated with sitting 3 or more hours per day.

After adjustment for factors associated with PA and sitting time, meeting US PA recommendations among mobile phone users was associated with being male (odds ratio [OR] 1.42, 95% CI 1.16-1.74), texting (OR 1.26, 95% CI 1.03-1.56), and having a higher acculturation score (OR 1.27, 95% CI 1.07-1.52), and it was inversely associated with being a former alcohol drinker (OR 0.71, 95% CI 0.57-0.87; Table 4). Sitting 3 or more hours per day among mobile phone users was associated with being male (OR 1.26, 95% CI 1.02-1.56), obese (OR 1.30, 95% CI 1.11-1.54), born in the United States (OR 2.08, 95% CI 1.64-2.62), higher education (OR 1.51, 95% CI 1.26-1.82), and being a former alcohol drinker (OR 1.48, 95% CI 1.12-1.95; Table 4). For mobile phone nonusers, being married (OR 2.01, 95% CI 1.09-3.68) was associated with meeting US PA recommendations, and sitting 3 or more hours per day was associated only with being born in the United States (OR 2.56, 95% CI 1.05-6.21; Table 5).

**Table 1:** Comparing mobile phone users (n=2556) with mobile phone nonusers (n=328) in the Mexican American Mano a Mano cohort.

Variable		Mobile phone users, N (%)	Mobile phone nonusers, N (%)	*P* value
**Age, years**							
	21-39	651 (25.5)	44 (13.4)	<.001
	40-54	1142 (44.7)	119 (36.3)	
	55+	763 (29.9)	165 (50.3)	
**Sex**							
	Male	738 (28.9)	85 (25.9)	.264
	Female	1818 (71.1)	243 (74.1)	
**Birthplace**							
	Mexico	1977 (77.4)	286 (87.2)	<.001
	United States	576 (22.6)	42 (12.8)	
**Marital status**							
	Not married	646 (25.3)	79 (24.1)	.638
	Married	1909 (74.7)	249 (75.9)	
**Education**							
	Some high school or less	1583 (62.0)	257 (78.4)	<.001
	High school graduate	972 (38.0)	71 (21.6)	
**Obesity**							
	Not obese	1170 (46.8)	167 (53.4)	.029
	Obese	1330 (53.2)	146 (46.6)	
**Acculturation**							
	Low	1277 (50.0)	239 (72.9)	<.001
	High	1279 (50.0)	89 (27.1)	
**Alcohol drinking status**							
	Current	597 (23.4)	53 (16.2)	.016
	Former	337 (13.2)	44 (13.4)	
	Never	1622 (63.5)	231 (70.4)	
**Smoking status**							
	Current	311 (12.2)	31 (9.5)	.313
	Former	472 (18.5)	51 (15.6)	
	Never	1772 (69.3)	245 (47.9)	
**Use text messaging**							
	Yes	1804 (70.6)	---	---
	No	749 (29.3)	---	
**Physical activity recommendations**							
	Not meeting	1583 (61.9)	228 (69.5)	.008
	Meeting	973 (38.1)	100 (30.5)	
**Sitting 3 or more hours/day**							
	Yes	1296 (50.7)	191 (58.2)	.011
	No	1259 (49.3)	137 (41.8)	

**Table 2:** Unadjusted odds ratios (95% CI) for mobile phone users for meeting physical activity recommendations and sitting 3 or more hours per day (n=2556).

Variable	Meeting PA^a^ recommendations		Sitting 3+ hours/day	
	OR^b^	95% CI	OR	95% CI
**Age**
Age 21-39 years	1.00		1.00	
Age 40-54 years	0.82	0.68-0.99	0.85	0.70-1.02
Age 55+ years	0.65	0.53-0.79	0.98	0.81-1.20
**Gender**
Female	1.00		1.00	
Male	1.49	1.25-1.77	1.38	1.16-1.64
**Country of origin**
Mexico-born	1.00		1.00	
US-born	1.04	0.86-1.27	2.74	2.25-3.33
**Marital status**
Not married	1.00		1.00	
Married	1.13	0.94-1.36	0.84	0.70-0.99
**Education**
Some HS^c^ or less	1.00		1.00	
HS graduate	1.21	1.03-1.43	1.85	1.57-2.17
**Weight status**
Not obese	1.00		1.00	
Obese	0.88	0.75-1.04	1.36	1.16-1.60
**Acculturation status**
Low acculturation	1.00		1.00	
High acculturation	1.42	1.21-1.66	1.81	1.55-2.12
**Alcohol status**
Never drinker	1.00		1.00	
Former drinker	0.93	0.73-1.20	1.79	1.41-2.27
Current drinker	1.76	1.46-2.13	1.60	1.33-1.94
**Smoking status**
Never smoker	1.00		1.00	
Former smoker	1.06	0.86-1.31	1.31	1.07-1.60
Current smoker	1.25	0.98-1.60	1.77	1.39-2.27
**Text messaging**
Do not use text messaging	1.00		1.00	
Use text messaging	1.39	1.16-1.66	1.31	1.10-1.55

^a^PA: physical activity.

^a^OR: odds ratio.

^a^HS: high school.

**Table 3:** Unadjusted odds ratios (95% CI) for mobile phone nonusers for meeting physical activity recommendations and sitting 3 or more hours per day (n=328).

Variable	Meeting PA^a^ recommendations		Sitting 3+ hours/day	
	OR^b^	95% CI	OR	95% CI
**Age**
Age 21-39 years	1.00		1.00	
Age 40-54 years	1.03	0.50-2.11	1.15	0.57-2.34
Age 55+ years	0.56	0.28-1.14	1.17	0.59-2.31
**Gender**
Female	1.00		1.00	
Male	1.17	0.69-1.98	2.10	1.28-3.47
**Country of origin**
Mexico-born	1.00		1.00	
US-born	0.90	0.44-1.84	4.18	2.05-8.52
**Marital status**
Not married	1.00		1.00	
Married	2.01	1.09-3.68	0.62	0.37-1.04
**Education**
Some HS^c^ or less	1.00		1.00	
HS graduate	1.55	0.89-2.68	1.84	1.08-3.12
**Weight status**
Not obese	1.00		1.00	
Obese	1.18	0.73-1.91	1.02	0.65-1.60
**Acculturation status**
Low acculturation	1.00		1.00	
High acculturation	1.41	0.84-2.37	2.55	1.55-4.19
**Alcohol status**
Never drinker	1.00		1.00	
Former drinker	1.03	0.51-2.08	0.40	0.20-0.77
Current drinker	1.48	0.80-2.77	0.96	0.52-1.76
**Smoking status**
Never smoker	1.00		1.00	
Former smoker	0.89	0.46-1.73	2.31	1.25-4.26
Current smoker	0.62	0.26-1.51	2.43	1.14-5.19

^a^PA: physical activity.

^a^OR: odds ratio.

^a^HS: high school.

**Table 4:** Multivariate adjusted odds ratios (95% CI) of mobile phone users for meeting physical activity recommendations and sitting 3 or more hours/day.

Variable	OR^a^	95% CI
Meeting PA^b^ recommendations		
	
Age 21-39 years	1.00	
Age 40-54 years	0.83	0.65-1.05
Age 55+ years	0.90	0.73-1.11
Female	1.00	
Male	1.42	1.16-1.74
Low acculturation	1.00	
High acculturation	1.27	1.07-1.52
Never alcohol drinker	1.00	
Former drinker	0.71	0.57-0.87
Current drinker	1.27	0.97-1.67
Do not use texting	1.00	
Use texting	1.26	1.03-1.56
Sitting 3 or more hours/day		
Female	1.00	
Male	1.26	1.02-1.56
Less than HS^c^	1.00	
HS graduate and beyond	1.51	1.26-1.82
Mexico-born	1.00	
US-born	2.08	1.64-2.62
Not married	1.00	
Married	0.99	0.82-1.21
Not obese	1.00	
Obese	1.30	1.11-1.54
Low acculturation	1.00	
High acculturation	1.07	0.89-1.30
Never drinker	1.00	
Former drinker	1.48	1.12-1.95
Current drinker	1.21	0.97-1.51
Never smoker	1.00	
Former smoker	1.03	0.82-1.29
Current smoker	1.24	0.95-1.63
Do not use texting	1.00	
Use texting	1.15	0.95-1.39

^a^OR: odds ratio.

^a^PA: physical activity.

^a^HS: high school.

**Table 5:** Multivariate adjusted odds ratios (95% CI) of mobile phone nonusers for meeting physical activity recommendations and sitting 3 or more hours/day.

Variable	OR^a^	95% CI
Meeting PA^b^ recommendations		
Not married	1.00	
Married	2.01	1.09-3.68
Sitting 3 or more hours/day		
Female	1.00	
Male	1.73	0.96-3.11
Mexico-born	1.00	
US-born	2.56	1.05-6.21
Low acculturation	1.00	
High acculturation	1.57	0.83-2.95
Never smoker	1.00	
Former smoker	1.48	0.73-3.01
Current smoker	1.26	0.53-2.97

^a^OR: odds ratio.

^b^PA: physical activity.

## Discussion

Our results highlight characteristics associated with mobile phone use and behavior in Mexican American adults, including age, birthplace, education, acculturation, and sex, and our study also reveals PA and sitting time differences between users and nonusers of mobile phones. In particular, mobile phone users were more obese, younger, from the United States, more educated, and reported more sitting time and PA than mobile phone nonusers. Meeting PA recommendations was associated with being male, using text messages, and higher acculturation. Although several studies have used mobile devices for increasing PA [4,5,9,10,23], few have examined the associations between mobile phones and behavior, and this study is among the first to quantify these associations among PA, sitting time, and various sociodemographic factors among users and nonusers of mobile phones in Mexican Americans.

In support of our findings, one national study of Hispanic people also reported that younger age and higher education were associated with mobile phone use [24], and the percentage of mobile phone users in our study was similar to that in other publications [7,11]. Lepp et al [8] showed that high frequency mobile phone use in college students was associated with less PA and more sedentary behavior. Although overall frequency of mobile phone use was not assessed in this study, mobile phone users among our participants were more likely to meet PA recommendations than nonusers and were less likely to sit 3 or more hours per day. Our findings are consistent with a study of Latina adolescents showing that mobile phone users report greater PA levels [25]. Additionally, one pilot study found that mobile phone use was associated with a decrease in the average number of daily minutes spent sitting in front of the television [26]. These results in combination indicate that mobile phones should be further studied to examine their potential for promoting PA and reducing sitting time in Hispanic populations.

Interestingly, factors associated with mobile phone use closely resemble those associated with acculturation. In particular, mobile phone users were more likely than nonusers to be obese, be more educated and have a higher acculturation score, and be born in the United States and live there longer, supporting results of previous research [24,25]. Other studies have shown that acculturation is positively associated with PA [16,26,27] and efforts to promote activity levels could be directed toward individuals with low acculturation scores. It is possible that acculturation might explain PA levels and mobile phone use in these participants. Further exploration is necessary to fully explain these differences.

Younger Hispanic adults are more likely to own a mobile device than older adults [7,24] and are also more likely to be physically active as found in this study, which may help explain differences among users and nonusers for meeting PA recommendations. More research is needed to determine why mobile phone users report less sitting time than nonusers.

In agreement with previous studies [16,17,28], males were more likely than females to meet PA recommendations and were also more likely to sit 3 or more hours per day. Our findings suggest that less than 40% of Mexican Americans meet US PA recommendations, which indicates this could be a potential target for a mobile phone intervention to improve health. Healthy People 2020 set a goal of 47.9% of US adults engaging in enough PA to meet recommendations [29], and research is needed to determine if mobile phones can be used to help reach this goal.

Mobile phone users who used text messaging were more likely to meet PA recommendations. It is not known how text messaging may influence domain-specific PA, such as leisure time or work activity; however, one pilot study in Latino adults found that an intervention providing daily text messages for 6 weeks resulted in an average increase of 146 minutes of exercise per week [30]. It is also possible that the mobile phone users possess smartphones that enable the users to increase their awareness and support for PA behavior through apps and Internet capabilities. One limitation of this study is that information on smartphone use (including mobile phone apps) and ownership was not collected, despite the increasing popularity and prevalence of this technology. Considering the wide array of PA apps available on smartphones and the fact that 64% of Hispanic people now own a smartphone [31,32], future research should examine the potential of these apps for helping Mexican American adults meet US PA recommendations.

Other limitations of this study include the cross-sectional and self-report nature of the data collected, which limits the ability to draw causal inferences and may subject the data to objectivity bias. Additionally, although the sitting time variable has acceptable reliability in adults, recent reports suggest that both men and women underreport their sitting time [33], which may have influenced results here. Furthermore, a single item assessing sitting time may not fully capture the entire spectrum in which an adult sits. Lastly, large portions of the data on media use of the mobile phones (eg, playing music or accessing the Internet) were missing and thus were not used in analyses. The media use data might be helpful for determining ways to promote PA using mobile phones, and future research should consider the best method for collecting this type of data.

In conclusion, this study identified associations between mobile phone use, text messaging, and PA in Mexican American adults, as well as behavioral differences between mobile phone users and nonusers. Mobile phone interventions have shown the ability to increase activity levels [4,5,9,10,34] and could certainly be tailored to promote PA in Mexican American mobile phone users. Future studies should examine the effects of text messaging for promoting domain-specific PA and determine ways to promote PA in those who do not use mobile phones because they are less active than mobile phone users.

## Abbreviations

BMI: body mass index
IPAQ: International Physical Activity Questionnaire
OR: odds ratio
PA: physical activity
US: United States
